# Synthesis of cyclophosphamide metabolites by a peroxygenase from *Marasmius rotula* for toxicological studies on human cancer cells

**DOI:** 10.1186/s13568-020-01064-w

**Published:** 2020-07-18

**Authors:** Susanne Steinbrecht, Jan Kiebist, Rosalie König, Markus Thiessen, Kai-Uwe Schmidtke, Sarah Kammerer, Jan-Heiner Küpper, Katrin Scheibner

**Affiliations:** 1 Institute of Biotechnology, Brandenburg University of Technology Cottbus-Senftenberg, Universitätsplatz 1, 01968 Senftenberg, Germany; 2 Fraunhofer Institute for Cell Therapy and Immunology, Branch Bioanalytics and Bioprocesses, Am Mühlenberg 13, 14476 Potsdam-Golm, Germany

**Keywords:** Biocatalysis, Cyclophosphamide, Human drug metabolites, Peroxygenase, Toxicity

## Abstract

Cyclophosphamide (CPA) represents a widely used anti-cancer prodrug that is converted by liver cytochrome P450 (CYP) enzymes into the primary metabolite 4-hydroxycyclophosphamide (4-OH-CPA), followed by non-enzymatic generation of the bioactive metabolites phosphoramide mustard and acrolein. The use of human drug metabolites as authentic standards to evaluate their toxicity is essential for drug development. However, the chemical synthesis of 4-OH-CPA is complex and leads to only low yields and undesired side products. In past years, fungal unspecific peroxygenases (UPOs) have raised to powerful biocatalysts. They can exert the identical selective oxyfunctionalization of organic compounds and drugs as known for CYP enzymes with hydrogen peroxide being used as sole cosubstrate. Herein, we report the efficient enzymatic hydroxylation of CPA using the unspecific peroxygenase from *Marasmius rotula* (*Mro*UPO) in a simple reaction design. Depending on the conditions used the primary liver metabolite 4-OH-CPA, its tautomer aldophosphamide (APA) and the overoxidized product 4-ketocyclophosphamide (4-keto-CPA) could be obtained. Using a kinetically controlled approach 4-OH-CPA was isolated with a yield of 32% (purity > 97.6%). Two human cancer cell lines (HepG2 and MCF-7) were treated with purified 4-OH-CPA produced by *Mro*UPO (4-OH-CPA^UPO^). 4-OH-CPA^UPO^–induced cytotoxicity as measured by a luminescent cell viability assay and its genotoxicity as measured by γH2AX foci formation was not significantly different to the commercially available standard. The high yield of 4-OH-CPA^UPO^ and its biological activity demonstrate that UPOs can be efficiently used to produce CYP-specific drug metabolites for pharmacological assessment.

## Key Points

- Unspecific peroxygenase from *Marasmius rotula* serves as efficient biocatalyst for selective cyclophosphamide hydroxylation.
- Peroxygenase-produced 4-hydroxycyclophosphamide can be used for direct cyto- and genotoxicity evaluation in human cancer cells.

## Introduction

Even though xenobiotic metabolism during biotransformation serves mostly as biochemical detoxification process, resulting metabolites might also cause adverse drug reactions and complications (Kirchmair et al. 2015; Park et al. 2011). Therefore, the synthesis of human drug metabolites (HDMs), especially of new drug candidates, plays an important role in pharmaceutical research and for the development of effective and safe drugs. HDMs are required as reference standards for structural confirmation and LC–MS recovery as well as for investigations of their pharmacological and toxicological properties in drug metabolism studies during preclinical safety assessment (Atrakchi 2009; FDA 2016; Schadt et al. 2018; Walker et al. 2009). Depending on the metabolite structure and chemistry involved, a classical chemical synthesis of HDMs can be very complicated, time and resource consuming (Atzrodt et al. 2012; Derdau et al. 2010).

The main pathway for metabolic clearance of pharmaceuticals is through oxidative mechanism predominantly catalyzed by liver cytochrome P450 monooxygenases (CYPs, EC 1.14.). They introduce in a highly selective manner oxygen into C-H-bonds of complex organic structures in order to convert lipophilic compounds into more hydrophilic and hence more excretable molecules (Guengerich 2008). The portfolio of methods to produce HDMs directly from the parental drug by regio and stereoselective oxyfunctionalization includes conventional oxidation (Chen and White 2010; Litvinas et al. 2009; Shan et al., 2012), biomimetic catalysis (Masood et al. 2012; Nicolas et al. 2013; Piera and Baeckvall 2008), electrochemical oxidation (Madsen et al. 2007; Nouri-Nigjeh et al. 2010) as well as microbial transformations (Amadio et al. 2013; Pervaiz et al. 2013; Sawayama et al. 2009; Schroer et al. 2010; Zollner et al. 2010). However, despite the versatility of those described methods the majority of these reactions are accompanied by low yields and selectivities and often lack also in scalability (Genovino et al. 2016; Zollner et al. 2010).

Unspecific peroxygenases (UPOs, EC 1.11.2.1) secreted by certain fungi have gained attention in the field of oxyfunctionalization. They represent a pronounced superfamily of heme thiolate proteins widespread in fungal kingdom that exhibit a promiscuity for oxygen transfer reactions by incorporating peroxide-derived oxygen into various organic substrates including unactivated hydrocarbons (Hofrichter et al. 2015; Hofrichter and Ullrich 2014; Kiebist et al. 2019). In contrast to membrane-bound, poorly stable and cofactor dependent P450 monooxygenases, secreted UPOs do not require complex cofactors like NAD(P)H or electron-transport systems (flavin-reductases, ferrodoxins) but solely hydrogen peroxide (Hofrichter et al. 2010). The first UPO was isolated from the basidiomycetous fungus *Agrocybe aegerita* in 2004 (Ullrich et al. 2004). In the following years further representatives were found i.a. in *Marasmius rotula* and *Chaetomium globosum* (Gröbe et al. 2011; Kiebist et al. 2017). Next to the handful of isolated and well characterized wild-type UPOs more than 5000 putative UPO sequences have been found in fungal genomes. However, their heterologous expression still seems to be quite difficult to realize and so far successful recombinant expression was only demonstrated in few cases such as in *Saccharomyces* and *Pichia*. The limiting steps might be the complex folding and formation of disulfide bridges (Molina-Espeja et al. 2014, 2015). However, it was shown that UPO-catalyzed reactions generally resemble those of CYPs including hydroxylation, epoxidation, dealkylation, heteroatom oxygenation, C–C fission, halide oxidation as well as one-electron oxidation analogously to classic peroxidases (Kiebist et al. 2019). These properties qualify UPOs as an appropriate and convenient tool for the preparative and sustainable production of HDMs as it was demonstrated in a panoply of examples (Gomez de Santos et al. 2018, 2019; Kiebist et al. 2015; Poraj-Kobielska et al. 2013, 2011).

**Fig. 1:**
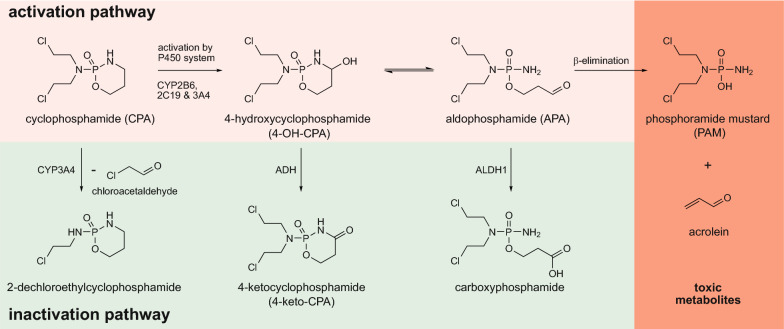
Metabolism of cyclophosphamide. The activation pathway requires hydroxylation of CPA by the human cytochrome P450 system (especially CYP2B6, -2C19 and -3A4). The primary metabolite 4-OH-CPA is in equilibrium with its tautomer aldophosphamide (APA) that undergoes $\beta$-elimination to the final toxic metabolites phosphoramide mustard (PAM) and acrolein. Through an oxidative reaction catalyzed by alcohol dehydrogenases (ADH) and particularly aldehyde dehydrogenase 1 (ALDH1), 4-OH-CPA and APA are irreversibly deactivated. Figure adapted from de Jonge et al. (de Jonge et al. 2005)

Here, we demonstrate the use of UPOs for metabolite synthesis of the well-known and commonly used cytostatic drug cyclophosphamide (CPA). CPA is used as alkylating agent belonging to the group of oxazaphosphorines and a classic example for a prodrug meaning it is inactive per se until it undergoes metabolic activation to achieve its anti-tumor activity (de Jonge et al. 2005; Fleming 1997; Rodriguez-Antona and Ingelman-Sundberg 2006). Various human CYP enzymes were shown to hydroxylate CPA resulting in phase I metabolite 4-hydroxycyclophosphamide (4-OH-CPA) that exists in equilibrium with its ring-opened tautomer aldophosphamide (APA). Those tautomers can either be converted into non-cytotoxic metabolites (4-ketocyclophosphamide, 4-carboxycyclophosphamide) or APA decomposes into phosphoramide mustard (PAM) by *β*-elimination of acrolein (Fig. 1). PAM is considered to be the primary metabolite responsible for the DNA-alkylating effect of CPA, while acrolein is a toxic by-product (Ahlmann and Hempel 2016; de Jonge et al. 2005). In the present work we aimed to produce 4-OH-CPA using UPOs on semi-preparative scale to evaluate the toxicity of those UPO-produced CPA metabolites directly on different human cancer cell lines.

## Materials and methods

### Material and reagents

4-Ketocyclophosphamide and perfosfamide were purchased from Niomech-IIT GmbH (Bielefeld, Germany). Cyclophosphamide was obtained from Alfa Aesar (Massachusetts, USA) and veratryl alcohol from Sigma-Aldrich (St. Louis, USA).

All chemicals were reagent-grade purity or analytical standards.

### Enzymes

Unspecific peroxygenases of *Agrocybe aegerita* (*Aae*UPO), *Coprinellus radians* (*Cra*UPO), *Marasmius rotula* (*Mro*UPO) and *Chaetomium globosum* (*Cgl*UPO) were produced and purified as previously described (Anh et al. 2007; Gröbe et al. 2011; Kiebist et al. 2017; Ullrich et al. 2004). The specific activities were 57, 23, 94 and 8.2 U mg^−1^ respectively, whereas 1 U represents the oxidation of 1 µmol 3,4-dimethoxybenzyl alcohol. Activities were measured by following the formation of veratraldehyde photometrically at 310 nm (*ε*_310_ = 9.3 mM^−1^ cm^−1^) in sodium phosphate buffer (50 mM, pH 7 and pH 5.5 for *Mro*UPO). The oxidation of veratryl alcohol (5 mM) was initiated by adding the cosubstrate hydrogen peroxide (2 mM). Protein content was measured with BCA assay using instructions of the manufacturer (Thermo Fisher Scientific, Germering, Germany). Glucose oxidase (GOx) from *Aspergillus niger* was purchased from Sigma-Aldrich (specific activity 215 U mg^−1^).

### UPO screening for CPA hydroxylation

In order to identify the most suitable UPO for preparation of 4-OH-CPA the four enzymes were incubated with CPA whereas a GOx/glucose system was applied for continuous hydrogen peroxide supply. The reaction mixtures (total volume 0.5 mL) contained 0.1 µM purified UPO in sodium acetate buffer (20 mM, pH 5.5) or BIS–Tris buffer (20 mM, pH 7.0) with CPA (1 mM) and α-d-glucose (2%, w/v). Reactions were started by addition of 1 nM GOx, stirred at 25 °C for 4 h and were stopped by the addition of 500 µL cooled acetonitrile (− 20 °C). The mixtures were centrifuged at 12,000 g for 10 min and subsequently analyzed by HPLC as described below. All reactions were performed in triplicate plus separated blanks without UPO and without GOx.

The HPLC system (VWR Hitachi) comprised L-2130 pump, L-220 autosampler, L-2300 column oven and L-2455 DAD coupled with a low temperature evaporative light scattering detector (ELSD 100, VWR, Radnor, PA, USA). Separation was performed on a Kinetex® column (C18, 5 µm, 100 Å, 150 × 4.6 mm, Phenomenex) with mobile phase A (diH_2_O) and B (acetonitrile) using following gradient at a flow rate of 1 mL min^−1^: 5% B at 0–2 min; 5–50% B at 2–20 min; 50% B at 20–22 min; 50–5% B at 22–24 min and 5% B at 24–26 min. Analytes were identified using authentic standards and high-resolution mass spectrometry (HRMS). Commercially available compound perfosfamide (PPA, 4-hydroperoxycyclophosphamide) served as standard for 4-OH-CPA. PPA was reduced by addition of sodium thiosulphate beforehand as given by manufactures instructions (Niomech—IIT GmbH, Bielefeld, Germany).

Chromatographic separation for LC–MS experiments were performed on a Thermo Scientific Vanquish Flex Quaternary UHPLC system (Thermo Fisher Scientific). The chromatographic column, eluents and gradient applied were the same as described before. The flow rate was 0.5 mL min^−1^. For high-resolution mass spectrometry (HRMS), a Thermo Scientific Q Exactive Plus quadrupole-Orbitrap mass spectrometer (Thermo Electron, Bremen, Germany) coupled with a heated electrospray ionization source in positive mode was used. The tune operating parameters were as follows: the rate of sheath gas flow and auxiliary gas flow was 60 and 15 (arbitrary unit), respectively; spray voltage 4.0 kV; the temperature of capillary and auxiliary gas heater was 320 °C and 400 °C, respectively; high-resolution MS^n^ was operated at full scan with a mass range of *m*/*z* 150–1500 at a resolution of 70,000 (*m*/*z* 200).

### Semi-preparative synthesis of 4-hydroxycyclophosphamide

Initially, to monitor the reaction course and to determine the optimal time to stop the CPA conversion in order to obtain a high yield of desired 4-OH-CPA and a reduced side product formation, the approach was carried out in 5-mL-scale. Therefore 13 mg CPA (0.05 mmol) were dissolved in sodium acetate buffer (20 mM, pH 5.5) and 1 µM *Mro*UPO was added. The reaction was started by adding the cosubstrate hydrogen peroxide by a syringe pump (Harvard Apparatus Pump11, 5 mM h^−1^) while stirring at 100 rpm and incubating at 25 °C. Samples (2 × 100 µL) were taken from the reaction mixture every 60 min; 100 µL were used to determine the remaining *Mro*UPO activity by veratryl alcohol assay (as described above), whereas the other 100 µL were diluted with 100 µL acetonitrile (− 20 °C), centrifuged for 10 min at 13,000 g and served to monitor metabolite formation by HPLC-ELSD.

Based on the results of the before mentioned approach the reaction was linearly scaled up to 100 mL-scale. Cyclophosphamide (261 mg, 1 mmol) was dissolved in sodium acetate buffer (20 mM, pH 5.5) with 1 µM *Mro*UPO, and the reaction was initiated by continuous supply of hydrogen peroxide (5 mM h^−1^). The reaction was stirred at 25 °C for 1 h. To stop the reaction 50 mL chloroform were added for 1 min. The phases were separated and the aqueous phase was extracted twice with chloroform (2 × 25 mL) and dried with Na_2_SO_4_. The aqueous phase was concentrated by column chromatography using C_18_-silica gel (90 Å, 230–400 mesh, Sigma-Aldrich). After elution of 4-OH-CPA with acetonitrile the solvent was removed under vacuum and subsequently subjected to preparative HPLC. Purification was conducted on a Shimadzu LC-20AP (Shimadzu, Japan) equipped with a FRC-10 A fraction collector, LC-20AP pump, SPD-10 A detector, and LiChrospher®100 column (LiCART®, 250 × 10 mm, RP-18, 10 µm, Merck KGaA, Darmstadt, Germany). Data analysis was performed using LC-2000 systems software. The injection volume was 2 mL and the mobile phase consisted of deionized water (A) and acetonitrile (B), with a gradient elution program similar as described for analytical HPLC above using a flow of 5 mL min^−1^. Fractions were collected, cooled on ice and analyzed by HPLC-ELSD. 4-OH-CPA containing fraction were pooled, acetonitrile was removed under vacuum and the aqueous solution was frozen at − 80 °C and finally concentrated by lyophilization. Purification by preparative HPLC yielded in 89 mg (32%) of a white powder with a purity of 97.6% (HPLC-ELSD).

HRMS (ESI^+^): *m*/*z* calculated for C_7_H_16_Cl_2_N_2_O_3_P [M + H]^+^: 277.0276, found: 277.0268 [M + H]^+^ (100%); *m*/*z* calculated for C_7_H_14_Cl_2_N_2_O_2_P [M + H-H_2_O]^+^: 259.0169, found: 259.0163 [M + H-H_2_O]^+^ (90%).

Stability of the synthesized 4-OH-CPA by *Mro*UPO (4-OH-CPA^UPO^) was examined in aqueous dilutions which were kept at 37 °C for 24 h or at − 80 °C for 4 weeks. Samples were analyzed by HPLC-ELSD.

### Cell culture

Human breast adenocarcinoma (MCF-7; ATCC: HTB-22, Manassas, USA) and human hepatoblastoma (HepG2; ATCC: HB-8065) cells were cultivated in Dulbecco’s minimal essential medium (DMEM) (Biochrom AG, Berlin, Germany) supplemented with 10% fetal bovine serum (Biochrom AG) and 2 mM l-glutamine (PAA Laboratories GmbH, Pasching, Austria) at 37 °C and 5% CO_2_ in a humidified incubator. For the experiments described we used passages 14–21 for HepG2 and 13–33 for MCF-7 cells.

### CellTiter-Glo®2.0 assay

HepG2 and MCF-7 cells were seeded at a density of 1.5 × 10^4^cells per well in 96-well plates (Sarstedt AG & Co). After 24 h, cells were treated with ten different concentrations of cyclophosphamide (CPA), perfosfamide (PPA) or 4-OH-CPA^UPO^ at a range of 0–1.6 mM for HepG2 cells and 0–400 µM for MCF-7 cells in standard medium. We found in previous experiments these concentration ranges to be optimal for EC_50_ determination for both cell lines. 24 h after substance treatment, evaluation of cell viability was performed using CellTiter-Glo®2.0 assay (Promega, Madison; USA) according to the manufacturer´s protocol. In short, substance-containing medium was replaced by 100 µL reaction mixture (50 µL fresh medium supplemented with 50 µL ATP reagent solution). Plates were shaken at 300 rpm for 2 min and then incubated for 10 min at room temperature in the dark. Lysates were transferred to a white-walled 96-well plate and luminescence signals were measured using FLUOstar Omega microplate reader (BMG Labtech, Ortenberg, Germany). The EC_50_ values were derived from three separate and distinct experiments, whereby the repetitions within each experiment were averaged. Statistical data analysis and nonlinear regression for determination of EC_50_ values (half maximum effective concentration) were performed using GraphPad Prism 6.0 (GraphPad Software Inc., San Diego, CA, USA). The Mann–Whitney U test was used to compare the EC_50_ values. *P* value < 0.05 was considered statistically significant.

### Detection of γH2AX foci

HepG2 (1.5 × 10^4^) and MCF-7 (2.5 × 10^4^) cells were seeded onto glass cover slips in 24-well plates (Sarstedt AG & Co). After 24 h, cells were treated with three different concentrations of CPA, PPA or 4-OH-CPA^UPO^ at a range of 0–50 µM for HepG2 cells and 0–25 µM for MCF-7 cells in standard medium. 24 h after substance treatment, cells were fixed in 4% paraformaldehyde (Merck Millipore, Massachusetts, USA) for 10 min, washed with PBS and permeabilized in 0.25% Triton X-100 (Carl Roth GmbH) for 3 min. After washing with PBS, cells were incubated for 1 h at RT with anti-phospho-Histone H2A.X (Ser139) mouse monoclonal IgG (clone JBW301, Cat. No. 05–636, Merck Millipore) diluted 1:1000 in PBS containing 1% BSA. After washing, cells were incubated for 1 h at RT with polyclonal Cy3-conjugated goat-anti-mouse antibody (Jackson ImmunoResearch Laboratories, Inc., West Grove) diluted 1:200 in PBS containing 1% BSA and 0.2 µg mL^−1^ DAPI (Carl Roth GmbH). Immunofluorescence was evaluated with an Olympus IX81 fluorescence microscope (Olympus, Tokyo, Japan).

## Results

### Human CPA metabolites produced by *Mro*UPO

Four unspecific peroxygenases secreted by different fungi were tested for their ability to selectively oxyfunctionalize cyclophosphamide (CPA). The reaction mixtures were analyzed by HPLC-ELSD and the formed products determined by HRMS using authentic standards of 4-ketocyclophosphamide (4-keto-CPA) and reduced perphosphamide (PPA). The latter is a peroxidized CPA derivative that can be reduced to 4-OH-CPA using sodium thiosulphate (Hilton, 1984).

**Fig. 2:**
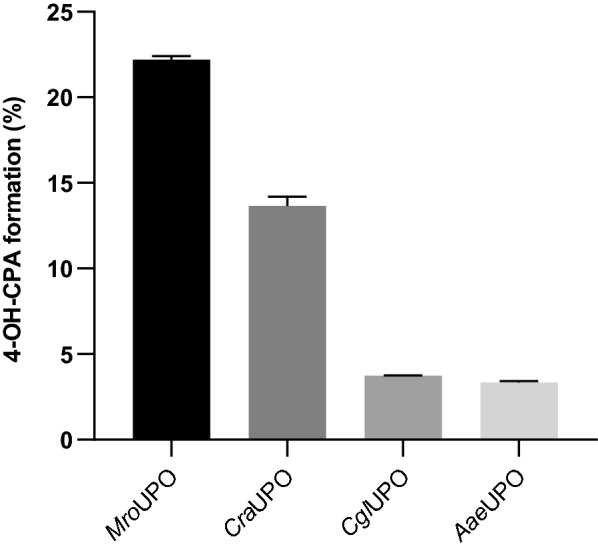
UPO screening for 4-OH-CPA production. The diagram displays the yield of 4-OH-CPA produced by different wild-type UPOs within 4 h using following conditions: 0.1 µM purified UPO, 1 nM GOx, 1 mM CPA, 2 wt% glucose, pH 5.5 (in case of *Mro*UPO) or 7.0 and 25 °C. The data represent mean values of three independent approaches with standard error of the mean (SEM)

**Scheme 1:**
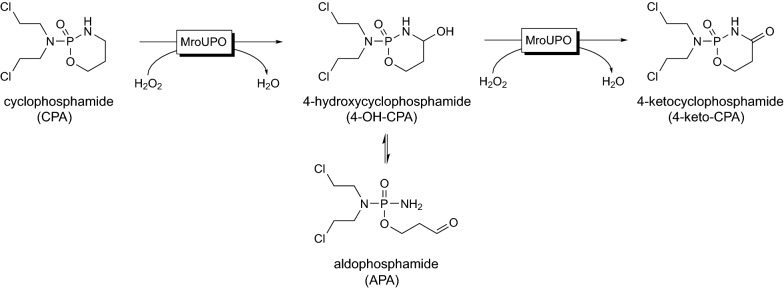
Cyclophosphamide converted by *Mro*UPO to human drug metabolites. *MroUPO* transformed of cyclophosphamide (CPA) into the intrinsic active metabolite 4-hydroxy-cyclophsophamide (4-OH-CPA), its tautomer aldophosphamide (APA) and the overoxidized side product 4-ketocyclophsophamide (4-keto-CPA)

All UPOs tested were able to hydroxylate CPA to form 4-OH-CPA, but with distinctly different yields. The most promising biocatalyst for the target reaction was *Mro*UPO (22%) followed by *Cra*UPO (14%), *Cgl*UPO (4%) and *Aae*UPO (3%) (Fig. 2). Other than the following products were not found with the tested enzymes. Along with the formation of 4-OH-CPA by *Mro*UPO (HRMS calcd. for C_7_H_16_Cl_2_N_2_O_3_P [M + H]^+^: 277.0276, found 277.0269) also its tautomer aldophosphamide (APA) and to a minor extend the side product 4-ketocyclophosphamide (4-keto-CPA, HRMS calcd. for C_7_H_14_Cl_2_N_2_O_3_P [M + H]^+^: 275.0119, found 275.0111) could be detected indicating an overoxidation of 4-OH-CPA (Scheme 1). Since 4-OH-CPA and APA equilibrate with each, the term 4-OH-CPA denote both compounds.

### Kinetically controlled enzymatic synthesis of 4-OH-CPA

**Fig. 3:**
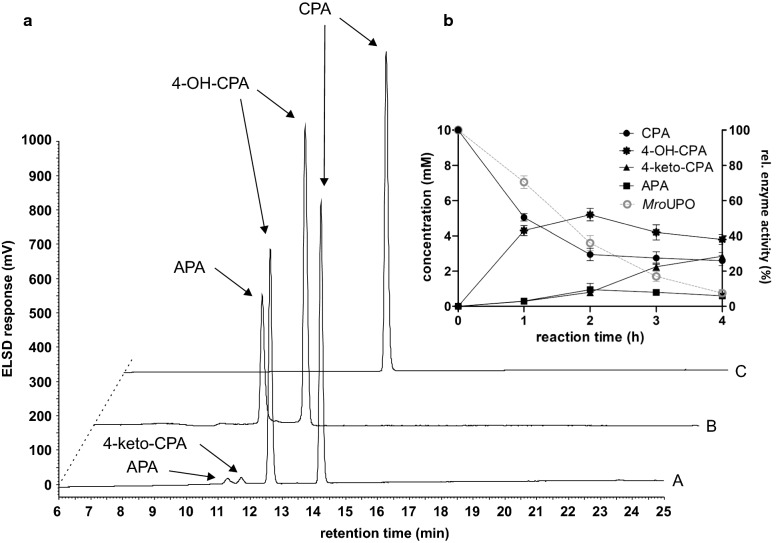
Time course of *Mro*UPO-catalyzed CPA conversion. **a** HPLC-ELSD elution profiles of (**A**) reaction mixture after 60 min (**B**) standards of aldophosphamide (APA) and 4-hydroxycyclophosphamide (4-OH-CPA) as products of perphosphamide (PPA) reduction and (**C**) standard of cyclophosphamide (CPA). **b** Time course of the CPA conversion by *Mro*UPO in 5 mL-scale using 10 mM CPA, 1 µM purified enzyme and 5 mM h^−1^ H_2_O_2_ in sodium acetate buffer (20 mM, pH 5.5) at 25 °C

Because of its superior metabolization activity of CPA, further investigations for 4-OH-CPA production was carried out with *Mro*UPO. In a small-scale approach (5 mL total volume) different parameters were varied including substrate–catalyst ratio and hydrogen peroxide supply (data not shown). The best conditions were used to follow the course of the metabolite formation by HPLC-ELSD indicating a high influence to the metabolite ratio by the reaction time (Fig. 3b). At the beginning, mainly 4-OH-CPA was formed (45%) and at a small extend also APA and 4-keto-CPA (Fig. 3a-A). Already after 2 h reaction time, the yield of 4-OH-CPA reached its maximum around 52% (turnover frequency (TOF) 3,000 h^−1^) while 10% APA and 4-keto-CPA were present, respectively. As the reaction proceeded, the concentration of 4-keto-CPA increased significantly within the next two hours, whereby the ratio of 4-OH-CPA and APA remained constant resulting in a total turnover number (TTN) value for C4-oxidation of about 10,000.

Since the main objective was to produce the primary CPA metabolite 4-OH-CPA by selective incorporation of oxygen using *Mro*UPO, a reaction time of 60 min was chosen for the semi-preparative scale based on the results given before. The reaction was stopped by adding chloroform yielding in 42% 4-OH-CPA and a small fraction of the side product 4-keto-CPA. To obtain the primary metabolite 4-OH-CPA at high purity, the reaction mixture was extracted, concentrated and purified by preparative HPLC. The purified fractions were further analyzed by HPLC-ELSD, accordingly pooled and finally the solvent was removed. As final product we achieved approximately 89 mg white solid of tautomers 4-OH-CPA and APA with a purity of 97.6% (HPLC-ELSD).

### 4-OH-CPA^UPO^ affects human cancer cell lines as its synthetic counterpart PPA

**Fig. 4:**
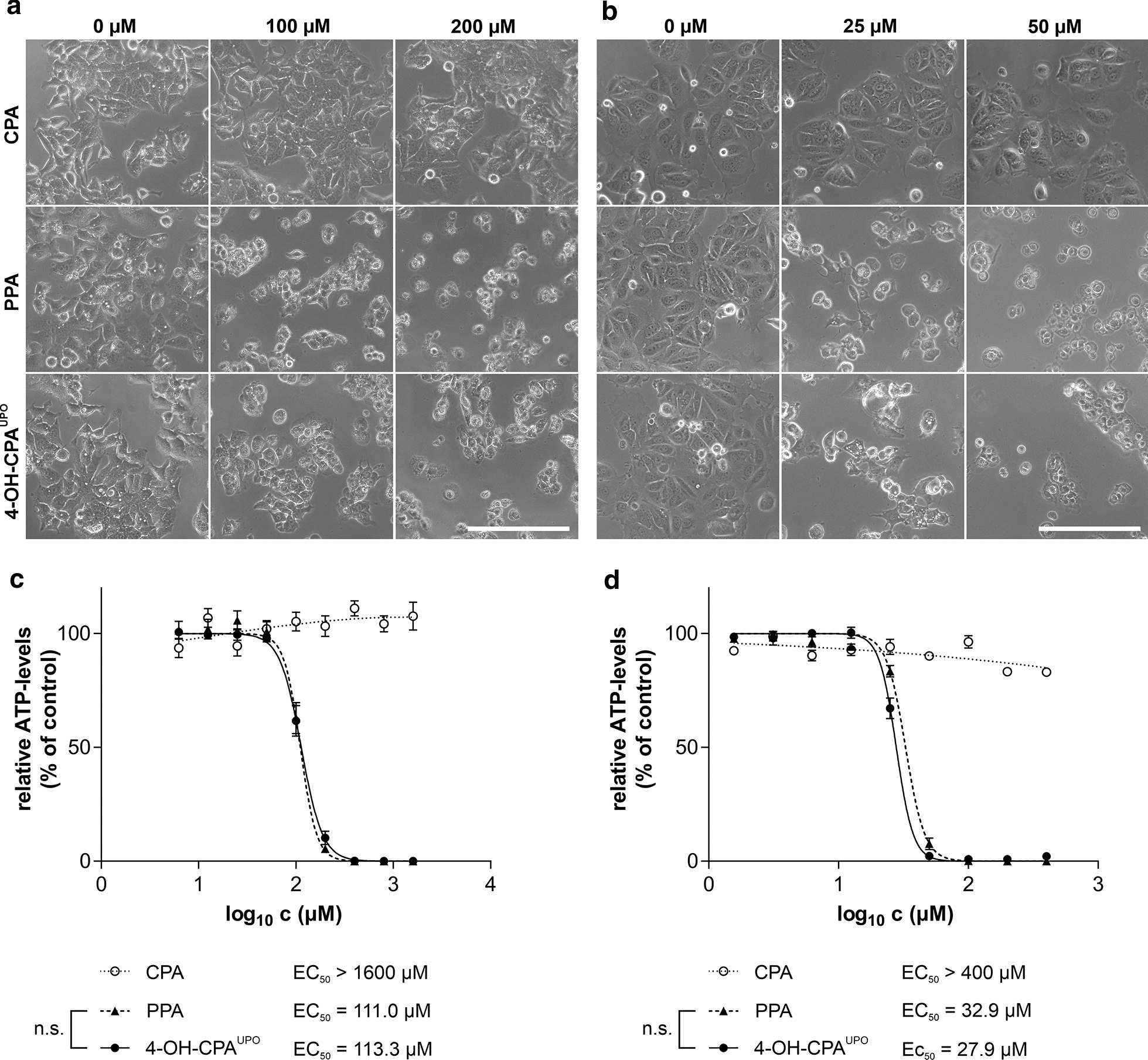
Morphology and metabolic activity of HepG2 and MCF-7 cells treated with CPA, PPA or 4-OH-CPA^UPO^. Cells were treated with different concentrations of the compounds for 24 h. Phase contrast images of (**a**) HepG2 and (**b**) MCF-7 cells were obtained with microscope CKX41 and 20 × objective (scale bar 200 µm). Relative viabilities of treated (**c**) HepG2 and (**d**) MCF-7 cells were evaluated by CellTiter-Glo®2.0 assay (as ATP levels). Data represent mean values ± SEM relative to untreated controls of three independent experiments in triplicates. Calculated EC_50_ values are indicated; n.s.: not significant

According to literature, 4-OH-CPA is in equilibrium with APA and decomposes into the cytotoxic compounds PAM and acrolein (Fig. 1) (de Jonge et al. 2005). While the therapeutic anti-cancer effect of CPA is attributed to the DNA-crosslinking metabolite PAM, acrolein causes toxic side effects through the production of highly reactive oxygen free radicals (Omole et al. 2018). Before incubation with cells, we tested by HPLC analysis the stability of *Mro*UPO-produced 4-OH-CPA (4-OH-CPA^UPO^) at different temperatures. While 4-OH-CPA^UPO^ decayed almost completely at 37 °C within 24 h, the metabolite was stable at − 80 °C for at least one month after purification (data not shown). Therefore, 4-OH-CPA^UPO^ was kept frozen at − 80 °C and thawed shortly before starting the cell experiments. We tested the biological effect of 4-OH-CPA^UPO^ on metabolic activity of two different human cancer cell lines, HepG2 and MCF-7. We exposed those cell lines to different concentrations of purified 4-OH-CPA^UPO^, the commercial standard PPA and unconverted CPA for 24 h. PPA decomposes rapidly into 4-OH-CPA in aqueous solution at 37 °C and its effects were therefore directly compared to those of 4-OH-CPA^UPO^ (Low et al. 1982). Microscopic examination showed that the cell morphology of both cell lines changed dramatically after treatment with 4-OH-CPA^UPO^ or PPA (Fig. 4a, b, middle and lower panels). While HepG2 and MCF-7 cells appeared unaltered after respective CPA treatment (Fig. 4a, b, upper panels), the cells exposed to the metabolite were rounded and detached from the cell culture flask as expected for cytotoxic compounds.

To compare the cytotoxic effects of all three compounds on human cancer cells, we performed a standard ATP assay and calculated EC_50_ values. CPA, up to the highest concentration used, did not result in any (HepG2) or only a slight (MCF-7) reduction of relative ATP levels (Fig. 4c, d). In contrast, treatment with 4-OH-CPA^UPO^ and commercial PPA led to complete ATP depletion in both cell lines at higher concentrations, indicating cell death. There was no significant difference between EC_50_ values of 4-OH-CPA^UPO^ and PPA in both cell lines. EC_50_ values for HepG2 were between 111 and 113 µM (Fig. 4c), while MCF-7 cells responded more sensitively to 4-OH-CPA^UPO^ and PPA treatment with lower EC_50_ values of 28 and 33 µM, respectively (Fig. 4d).

**Fig. 5:**
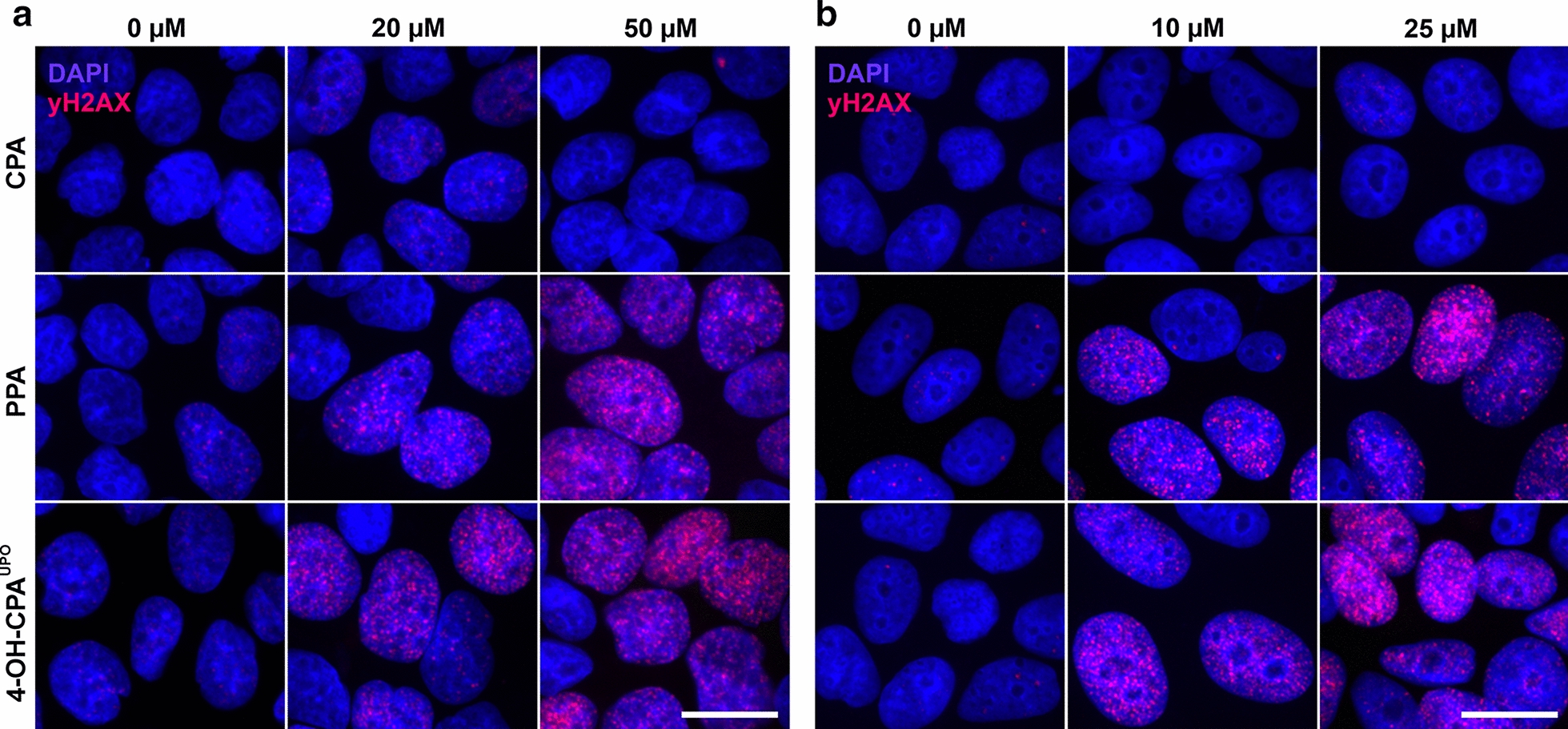
Genotoxicity of CPA, PPA or 4-OH-CPA^UPO^ on HepG2 and MCF-7 cells. Cells were treated with different concentrations of the respective compounds for 24 h. γH2AX foci formation was determined using indirect immunofluorescence (pink). Nuclei were stained with DAPI (blue). Representative images of (**a**) HepG2 and (**b**) MCF-7 nuclei are shown as obtained with Olympus IX81 fluorescence microscope and 100 × objective (scale bar 5 µm)

To directly analyze genotoxicity of 4-OH-CPA^UPO^, we investigated the formation of γH2AX foci using indirect immunofluorescence. After exposure of cancer cells to CPA, 4-OH-CPA^UPO^, as well as the PPA in concentrations up to 50 µM for HepG2 and 25 µM for MCF-7 for 24 h, the phosphorylation of H2AX (γH2AX), seen as fluorescent foci in cell nuclei, was measured. Cells treated with increasing concentrations of prodrug CPA did not show any significant formation of γH2AX foci (Fig. 5a, b, upper panels). In contrast, nuclei of both cell lines showed a clear concentration-dependent increase in γH2AX foci formation after treatment with PPA and 4-OH-CPA^UPO^ (Fig. 5a, b, middle and lower panels).

## Discussion

The U.S. Food and Drug Administration (FDA) clearly recommends the identification and characterization of drug metabolites for nonclinical safety assessments during early drug development prior to the first human studies (FDA 2016). Usually, standard animal experiments for toxicological drug testing are used in these nonclinical safety evaluations. However, the metabolite profiles can differ from species to species both qualitatively and quantitatively. Therefore, the identification of major human drug metabolites (HDMs) and approaches for their synthesis are essential in order to use them for further in vitro and in vivo evaluations of their pharmacological and toxicological consequences (Baillie et al. 2002; FDA 2016; Nedderman 2009). An efficient approach in the production of drug metabolites on a preparative scale is the use of native human CYPs as biocatalysts (Bernhardt and Urlacher 2014). The range extends from whole cell systems such as CYP-overexpressing human cell lines, recombinant expression of individual human CYP isoforms in different microorganisms up to isolated liver microsomes (Schroer et al. 2010; Steinbrecht et al. 2019a). Since the use of whole cell systems is restricted, for instance, by low expression levels of certain CYP isoforms and limitations in substrate uptake and product transport, simplified biocatalysis using isolated enzymes have been developed (Schroer et al. 2010; Urlacher and Girhard 2012). Besides the use of cytosolic bacterial CYP enzymes, in the recent years the enzyme class of unspecific peroxygenases (UPOs) has proven to be a large activation toolbox for several pharmaceutical agents due to their metabolic similarities to human cytochrome P450 enzymes. Successful drug conversion by UPOs into their known metabolites was demonstrated for example with diclofenac, ibuprofen, propranolol, naproxen, sildenafil, volixibat and testosterone (Kiebist et al. 2019, 2015, 2017; Kinne et al. 2009; Poraj-Kobielska et al. 2011).

Here, we were interested to go a significant step forward, i.e. to compare the biological effects on human target cells of an UPO-produced metabolite with its commercially available synthetic counterpart. For this we studied the conversion of cyclophosphamide, a model drug for bioactivation, by selective hydroxylation at C-4 catalyzed by an UPO secreted from agaric fungus *Marasmius rotula* (*Mro*UPO). HPLC-ELSD analyses and comparison with commercial standards showed an enzymatic conversion of CPA resulting in a time-dependent formation of the main product 4-hydroxycyclophosphamide (4-OH-CPA), the corresponding tautomer aldophosphamide (APA), and the overoxidized side product 4-ketocyclophosphamide (4-keto-CPA). CPA oxyfunctionalization by hydroxylation reflects the main activation pathway of CPA described in the literature, which is catalyzed in vivo by various human CYPs such as CYP2A6, 2B6, 3A4/5, 2C9, 2C8, 2C18 and 2C19 (Fig. 1) (Chang et al., 1993; Chang et al., 1997a, b; Ren et al., 1997; Xie et al., 2003). However, the use of human liver microsomes to convert CPA into its known human metabolites was shown to result in only in low yields (Chang, et al., 1993; Choi et al., 2015).

Synthetical production of 4-OH-CPA by conventional chemical methods dates back to the 1970s and describes the preparation of perfosfamide (PPA, 4-hydroperoxycyclophosphamide) as a precursor that intracellularly decays to 4-OH-CPA. PPA can be produced by Fenton oxidation of CPA, which uses Fe^2+^ ions as iron(II) sulphate and hydrogen peroxide in an acidic solution. This oxidation method achieved a yield of only 3–4% PPA and 11% 4-keto-CPA (van der Steen et al., 1973). Direct ozonation of cyclophosphamide or of *O*-3-butenyl-*N*,*N*-bis(2-chloroethyl)-phosphordamidate is another option for PPA synthesis in only one step. Ozonation of CPA gave a yield of 20% PPA and 50% 4-keto-CPA.(Hohorst et al., 1976) Takamizawa et al*.* further described an ozone-based method with a high PPA output of 50–60%, which resulted in about 40% 4-OH-CPA after deoxygenation (Takamizawa et al. 1975).

After 60 min of CPA conversion with *Mro*UPO, we achieved a 4-OH-CPA yield of 42% with less than 3% of the undesired by-product 4-keto-CPA. Thus, the enzymatic method presented here using unspecific peroxygenases reveals to be competitive with conventional chemical methods concerning the yield of 4-OH-CPA. Regarding the low amount of unwanted 4-keto-CPA, the reaction catalyzed by *Mro*UPO has even proved to be superior. In addition, due to the enzymatic conversion under mild conditions, instead of using aggressive oxidants, the regioselectivity of hydroxylation is more precise and no special safety instructions are required during performance.

Purified 4-OH-CPA^UPO^ was used to treat the cancer cell lines HepG2 and MCF-7 for re-evaluation of CPA and its metabolite toxicity. Since 4-OH-CPA is known to be very unstable with a half-life of only a few minutes in plasma at 37 °C, we were interested in metabolite stability under the experimental conditions we used (de Jonge et al. 2005; Johansson and Bielenstein 1994). In stability analysis of 4-OH-CPA^UPO^ we detected an almost complete degradation after 24 h at 37 °C in aqueous solution (data not shown). At − 80 °C, however, 4-OH-CPA^UPO^ remained stable for at least one month, so storage at this temperature was recommended.

In this study we aimed to compare the cytotoxic effect of 4-OH-CPA^UPO^ on human cancer cells with that of commercially available PPA and the parental substance CPA using a 24 h incubation period. PPA exhibits higher stability and is often used as an alternative to 4-OH-CPA. It can be equated with the use of 4-OH-CPA due to its rapid degradation into 4-OH-CPA in aqueous solution and has the same toxicity (Hales 1982; Hohorst et al. 1976; Johansson and Bielenstein 1994; Peter and Hohorst 1979). Low et al*.* described that PPA is converted to 4-OH-CPA at pH 7.4 and 37 °C (corresponds to cell culture conditions) with a t_1/2_ of 43 min (Low et al. 1982).

CPA is commonly used to treat various types of autoimmune and neoplastic diseases, including breast cancer. Since breast cancer cell line MCF-7 is commonly used to study antitumor drugs, it provides a suitable cell model for the assessment of CPA metabolite therapeutic effects (Kars et al. 2006; Li et al. 2001; Prados et al. 2010; Shui-Tein Chen 2002; Trebunova et al. 2012). HepG2 cells, on the other hand, were most frequently used as human liver cell model for toxicity studies and can contribute to the risk assessment of drug-induced liver injury (Brandon et al. 2003; Yokoyama et al. 2018). Although they exhibit negligible activity of most xenobiotic metabolizing enzymes, HepG2 cells retained several hepatocyte-specific characteristics (Javitt 1990; Knowles et al. 1980).

Dose dependently, short-term exposure (24 h) of HepG2 and MCF-7 cells to 4-OH-CPA^UPO^ or PPA resulted in a dramatic change in cell morphology and a complete reduction of metabolic activity (Fig. 4). Within the cell 4-OH-CPA decays to phosphoramid mustard (PAM), which is a bifunctional DNA alkylating agent causing intra- and interstrand cross-links and DNA double-strand breaks that can kill dividing cells. It is well known that DNA double-strand break formation is accompanied by phosphorylation of histone H2AX (γH2AX) (Ganesan and Keating 2015; Scharer 2005). After immunofluorescence staining of emerging nuclear γH2AX foci in 4-OH-CPA^UPO^ and PPA-treated cells, the genotoxicity via PAM of those substances could be demonstrated (Fig. 5). With parental drug CPA, almost no toxic effect on cell morphology, metabolic activity or DNA double-strand break formation could be detected in the concentration range used. Therefore, basal expression of human CYPs involved in drug metabolism in HepG2 and MCF-7 cells seemed not to be sufficient for CPA activation (Guo et al. 2011; Mitra et al. 2011; Olsavsky et al. 2007).

Furthermore, a H2AX phosphorylation in the nuclei of both cell lines was already observed during treatment at the lower concentration range of 4-OH-CPA^UPO^ and PPA (Fig. 5). In contrast, the ATP assay showed no change in ATP levels at those concentrations (Fig. 4c, d). We recently found that such standard metabolic activity assays can dramatically underestimate the effects of genotoxic substances (Steinbrecht et al., 2019b). Therefore, the detection of γH2AX foci after drug treatment is recommended as an additional sensitive test to avoid false negative results during in vitro cytotoxicity analysis of genotoxic agents. Here we found that cytostatic and genotoxic effects of 4-OH-CPA^UPO^ on both human cancer cell lines was nearly identical to that of commercial PPA. This clearly demonstrate that HDMs produced by UPOs are suitable for in vitro toxicity tests.

Alcohol and aldehyde dehydrogenases (ADH, ALDH) are mainly involved in CPA detoxification by oxidizing CPA metabolites of the activation pathway to non-toxic by-products (Fig. 1). Especially ALDH enzymes are known to be distributed tissue-specifically and vary in their expression in different cell types (von Eitzen et al. 1994; Wang and Wang, 2012). Therefore, a different sensitivity to CPA and 4-OH-CPA has probably already been observed in several cells (Ohtani et al. 2006). Based on the EC_50_ values determined for 4-OH-CPA^UPO^ and PPA, we found a higher sensitivity in MCF-7 compared to HepG2 cells (Fig. 4c, d). Even if ALDH isotypes are expressed in both cell lines, it can be assumed that ALDH activity is higher in HepG2 than in MCF-7 cells due to its liver tissue origin (Ciccone et al. 2018; Crabb et al. 2004; Guo et al. 2011; Sladek et al. 2002). If this is true, more aldophosphamide could be deactivated to carboxyphosphamide resulting in higher resistance of HepG2 cells to CPA metabolites. It is also possible that variable intracellular phosphate levels affect the decay of 4-OH-CPA to PAM and acrolein (Low et al. 1982).

In summary, we identified UPO secreted by *Marasmius rotula* as an efficient biocatalyst for the reproducible and selective synthesis of the known CPA metabolite 4-OH-CPA at a semi-preparative scale. 4-OH-CPA^UPO^ could directly be used to evaluate its cyto- and genotoxicity in cell culture experiments using a standard metabolic activity assay on ATP levels and a γH2AX foci test to analyze DNA double-strand break formation. Via UPOs cost-effective and efficient production of relevant HDMs is possible as demonstrated here for 4-OH-CPA^UPO^ in only one simple enzymatic step. UPO-produced HDMs can thus serve as a suitable tool for metabolite risk assessment of already marketed or novel drug candidates in cell cultures or even in vivo. The large number of putative UPO sequences found in fungi and their broad catalytic spectrum represents a growing toolbox for future large-scale production of a wide range of HDMs.

## Data Availability

All data and materials have been included in the main article.
